# A global ‘Common Core’ framework for family medicine faculty development: Getting the right person, doing the right thing, right!

**DOI:** 10.4102/phcfm.v18i1.5371

**Published:** 2026-06-13

**Authors:** Esther M. Johnston, Ramakrishna Prasad, Bassim Birkland, Klaus B. von Pressentin, Sophie Watson, Annika Carlson, Shailendra Prasad

**Affiliations:** 1 The Global Engagement Network for Primary Health Care, Minneapolis, United States of America; 2 Department of Family Medicine and Community Health, Medical School, University of Minnesota, Minneapolis, United States of America; 3 Center for Global Health and Social Responsibility, University of Minnesota, Minneapolis, United States of America; 4 National Centre for Primary Care Research and Policy, Academy of Family Physicians of India, Bangalore, India; 5 Department of Family Medicine, University of Zambia, Lusaka, Zambia; 6 Seed Global Health, Boston, United States of America; 7 Department of Family, Community and Emergency Care, Faculty of Health Sciences, University of Cape Town, Cape Town, South Africa

**Keywords:** family medicine, family physicians, medical education, faculty development, clinical education

## Abstract

**Background:**

Family medicine is a medical speciality with a global reach, with common core principles but significant regional variations in training pathways, certification and recognition and clinical practice.

**Aim:**

Partly because of the differences in how family medicine is defined and practised in different areas, there are no existing globally informed guidelines on how to prepare family medicine faculty. This study aims to address this need.

**Setting:**

This study was conducted with an expert group composed of family medicine faculty development experts from across the world.

**Methods:**

A modified Delphi survey, grounded in a constructivist paradigm, was used to obtain consensus from family medicine faculty development experts representing a diverse range of geographical regions. We applied a combined approach to framework analysis, identifying themes deductively from an existing framework for general clinical educator development, as well as inductively from the comments of study participants. We analysed these responses using thematic analysis.

**Results:**

The final framework for family medicine faculty development includes three domains, encompassing the technical skills, knowledge and interpersonal approach, and professional role modelling expected of a family medicine educator.

**Conclusion:**

This study offers a novel framework for ensuring quality in the educators who are training the next generation of family medicine physicians.

**Contribution:**

Developed through consensus of a global expert team, this framework offers specific recommendations regarding technical skills, knowledge, philosophical approaches and professionalism, while offering sufficient flexibility to be applied in a wide array of regions and settings, reflecting the practice of family medicine itself.

## Introduction

A long history of general practice existed around the world when family medicine first emerged as a medical speciality in the 1960s.^1^ Over the last 50 years, the discipline has grown and expanded, and is now recognised as a distinct medical speciality in many countries.^2^ Family medicine plays a pivotal role in healthcare systems worldwide, providing comprehensive and patient-centred care across the lifespan.^3^ With its broad scope and focus on continuity of care, family medicine demands a unique skill set and a multifaceted approach to healthcare delivery. In some countries, the speciality is known as general practice, while in others, it is referred to as family medicine. In this article, we will utilise the term family medicine for both.^4^

In each country where it is practised, family medicine takes on a slightly different form in its training requirements and ultimate practice models. In countries where family medicine is recognised as a speciality, physicians spend 2–4 years in training after completing graduate studies. In other countries where family medicine is not formally recognised, generalist physicians are often regarded as family medicine physicians in their scope of practice. In some regions, family medicine is primarily an outpatient discipline, focusing exclusively on patient care through clinics. In others, family medicine provides surgical obstetrics and general surgical services within their communities.^2^ Even within the same nation, two family medicine physicians may have very different scopes of practice.

Family medicine encompasses all the tenets of primary care and, therefore, forms a foundational element in primary health care-based models aimed at achieving universal health coverage.^4,5^ As the importance and geographic reach of family medicine continue to grow, it is imperative to examine the foundational components that shape its quality and effectiveness, recognising the variations in ultimate practice. One critical aspect in this is the professional development of family medicine faculty – the educators and mentors responsible for shaping the next generation of family physicians.

Faculty development practices in family medicine, as in other medical specialties, hold the key to maintaining the highest standards of care, adapting to evolving healthcare needs, and advancing research and innovation. A few regional surveys have previously examined the resources for and roadblocks to family medicine faculty development.^6,7^ One previous literature review has summarised the specific activities that comprise family medicine faculty development globally.^8^ At least three countries offer faculty development models informed by their national contexts.^9,10,11^ The structures and outcomes of discrete faculty development programmes in family medicine within local and regional contexts have been published.^12,13^ Yet, many family medicine training programmes, as well as national and regional professional bodies, lack a framework to identify and respond to faculty development needs. The World Organization of Family Doctors (WONCA) has recognised the value of such frameworks in the past, developing global standards for undergraduate and postgraduate education, as well as continuing professional development in family medicine.^14,15,16^ These standards may serve as a scaffold that can be further adapted to meet the needs of individual national and regional contexts.

However, while there has been significant research into medical education and faculty development in various fields of medicine, there is not yet a comprehensive, global framework that specifically defines needed skills and approaches for educators in family medicine, recognising the variation that exists in clinical practice where graduates may go on to engage in different settings. Strengthening faculty development aligns with global and regional priorities for universal health coverage, as family medicine educators play a pivotal role in preparing clinicians to deliver comprehensive, community-based care.

This study aims to contribute to the body of knowledge that shapes the future of family medicine and its role in healthcare globally by establishing consensus on a common core of skills and principles in family medicine faculty development. It focuses on both the philosophical tenets and the technical components essential to family medicine educators.

## Research methods and design

### Study design and setting

We utilised a modified Delphi survey model, embracing a constructivist paradigm, to obtain consensus from experts from around the world.^17,18,19,20^

### Study population and sampling strategy

We applied discriminative snowball sampling to develop a list of family medicine faculty with experience and interest in faculty development to form an initial expert panel. We identified prominent family medicine educators and faculty developers from our professional networks. We also posted to the online forum for the WONCA Working Party on Education and contacted the Presidents of each WONCA region to ask for names of those considered ‘experts in family medicine development’. This approach aimed to identify a globally distributed group of experts in family medicine faculty development. We then pooled these names and cross-tabulated (to prioritise the most frequently referenced names) and narrowed to ensure at least two to four representatives from across six world regions, aligning with World Health Organization (WHO) regions: Africa, Americas, South-East Asia, Europe, Eastern Mediterranean and Western Pacific.

We then emailed those on the final list of names to invite them to participate in the study and to confirm that they met the inclusion criteria. Inclusion criteria were: family medicine faculty who self-identified mid-to-late career (having worked as a faculty member for more than 5 years), self-identified as being recognised in their country and/or region as someone who contributes significantly to family medicine faculty development, and/or as a faculty member who mentors early career colleagues, and/or occupies critical leadership roles, whether formally or informally. Participants were offered professional translation of the survey and its results in their preferred language.

### Data collection and analysis

We conducted a review of existing literature and created a summary to share with survey participants. We then developed an online survey using Qualtrics survey software (Provo, Utah), comprising both structured and open-ended questions to generate ideas and gather feedback on an existing model of clinical faculty development, utilising a ‘framework for developing excellence as a clinical educator’ by Hesketh et al.,^21^ with modifications based on literature review and author consensus to better capture the unique aspects of serving as a clinical educator in family medicine. The survey (Online Appendix 1) collected identifying information from the respondents, including names, to further ensure a diverse sample. However, responses to the rest of the survey questions were analysed anonymously in aggregate, and respondents were informed of this in the survey’s introduction.

During the first round of data collection, the questionnaire was distributed to the panel of experts. Three authors (Esther M. Johnston, Shailendra Prasad and Annika Carlson) used a framework analysis to identify themes deductively from the aforementioned framework, as well as inductively from the comments of study participants.^22^ We then applied thematic analysis to identify common themes. Based on review of existing literature regarding Delphi study methodology, a minimum threshold of 80% agreement was set.

Following this, the entire authorship group reviewed the results and developed a second online questionnaire (see Online Appendix 2) to share the anonymised first round of responses and request further feedback and/or insights. This questionnaire also included a proposed framework for family medicine faculty development. This was then emailed back to the initial group of respondents. Experts were asked to review and provide feedback on the model.

We analysed these responses to identify common themes and trends and determined that the predetermined level of consensus was achieved. No significant variation in opinion was observed, nor were there any new emergent themes with the second survey.

While quantitative data analysis is frequently employed in Delphi studies, our team recognises its role is often supplementary rather than critical for reporting key findings, because the primary objective of studies such as ours is to explore nuanced expert opinions and identify emergent themes captured by qualitative analysis.^23^ A reliance on quantitative metrics would therefore distract from a comprehensive understanding of expert convergence.^24^

With this in mind, we emphasise qualitative assessments of thematic saturation and consensus, rather than statistical significance.

### Ethical considerations

This research was reviewed and determined not to constitute human subjects research by the Human Research Protections Programme (HRPP) of the University of Minnesota Institutional Review Board (ID# STUDY00020533).

## Results

**FIGURE 1:**
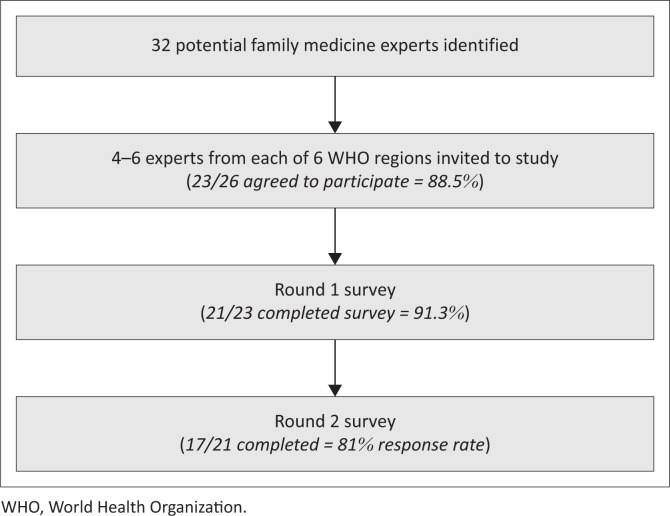
Delphi expert panel response rates.

An initial list of 32 family medicine faculty with experience or interest in faculty development was generated as described above. Four experts from each WHO region were invited to comprise an initial expert panel. If we had no initial response to our invitation or an invited expert declined, and we had another expert on our list from that region, we extended additional invitations. In total, 23 of those 26 (88.5%) invited agreed to participate in the study. Of those who initially agreed to participate in the Delphi panel, the response rate was 21 out of 23 (91.3%) to the first-round survey. Those 21 respondents were then invited to participate in the second round, and 17 of them (81%) responded (Figure 1). The demographics of the participants are shown in Table 1.

**TABLE 1:** Participant demographics.

Demographics	Accepted invitation to participate in Delphi study (*n*)	Completed first round survey (*n*)	Completed second round survey (*n*)
**Respondents by WHO region**			
Africa	4	4	4
Americas	4	4	4
Eastern Mediterranean	4	3	1
European	4	3	2
South-East Asia	4	4	4
Western Pacific	3	3	2
**Number of years of clinical practice following completion of family medicine postgraduate/residency training**			
6–10 years	-	3	2
11+ years	-	18	15

**Total number of participants (%)**	**23**	**21**	**17**

Note: Accepted invitation to participate in Delphi study: *N* = 26 (88.5%); Completed first round survey: *N* = 23 (91.3%); Completed second round survey: *N* = 21 (81%).

WHO, World Health Organization.

One participant requested a Spanish version of the survey, which we developed using a professional translator; responses were given in Spanish. The rest of the surveys were conducted in English.

In the first round of the Delphi study, participants were asked to prioritise skills, competencies and characteristics of a family medicine educator. Some items stood out as clearly higher priorities, and others as clearly lower, but the majority had no clear consensus on ranking. Based on the results of the first round of the Delphi, a proposed classification of some items in the framework was classified as higher/lower priority for round 2. However, at the end of round 2, no consensus was reached on how to prioritise various elements, and many respondents expressed that the prioritisation of elements in one setting might not accurately reflect the needs of another. Given this, prioritisation of different elements within the model was removed.

Additionally, participants in both rounds of the survey provided open-ended feedback regarding missing elements from the framework. Several of the overarching themes identified in the analysis, with representative quotes, are shown in Table 2. These themes were used to expand and modify the initial framework.

**TABLE 2:** Thematic analysis: Key elements respondents identified as essential to faculty development in family medicine.

Theme	Representative quotes
Grounding in the spirit of generalism	‘Mastering the principles and concepts of family medicine.’ (Round 1, Participant 13) ‘Understand principles of generalism, understand the larger socio-political context for learning clinical medicine and community health.’ (Round 2, Participant 11)
Community & societal awareness	‘Have high level of situational/contextual awareness, e.g. current social/political challenges that students and their communities face.’ (Round 1, Participant 21) ‘Family medicine educators should be able to teach and model cultural competence, recognising how culture, values and socio-economic factors significantly impact patient care. Educators must guide students in addressing healthcare disparities and adapting care to meet the unique needs of diverse populations, fostering an environment of inclusion and respect for all.’ (Round 2, Participant 10)
Knowledge and ability to navigate the health system	‘Need to understand the policy/legal framework within which the health system functions.’ (Round 1, Participant 21)
Operating in interdisciplinary teams	‘Teamwork: multi- and interdisciplinary collaboration.’ (Round 1, Participant 13) ‘Enable student participation in authentic work of clinical teams – foster student agency.’ (Round 1, Participant 10)
Ability to teach clinical governance and practice management	‘Needs to be able to provide clinical governance training.’ (Round 1, participant 21)
Emotional intelligence & interpersonal skills	‘Be open to having one’s weaknesses exposed. There is some good literature on this topic of balancing vulnerability with credibility.’ (Round 1, Participant 20) ‘Willingness to accept feedback and skills in receiving this.’ (Round1, Participant 20) ‘Appropriate use of humour is an essential role of the educator.’ (Round 1, Participant 20)
Personal development	‘Engage in ongoing reflection into personal strengths and weaknesses in clinical and community medicine – but rather higher priority for engage in ongoing reflection into personal development [*professional identity formation*] as a clinician-teacher.’ (Round 2, Participant 11)

After two rounds, agreement was reached that the final model (Table 3, Table 4 and Table 5) captures the key elements of skills, competencies and characteristics of a family medicine educator. When asked if this model would be helpful to the respondent in identifying faculty development needs among an educator team, 15 out of 17 (88.2%) said yes, 0 out of 17 said no, and 2 out of 17 (11.8%) of respondents were unsure.

**TABLE 3:** A model for family medicine faculty development: What the family medicine educator is able to do.

Level	What the family medicine educator is able to do ‘Doing the right thing’ Technical skills								

Level 1	Knowledge of systems & principles			Interpersonal skills & professional approach	Analytical & research skills				
Level 2	A –Plan learning	B – Develop and work with learning resources	C – Facilitate and manage learning	D – Teach large and small groups	E – Teach in a clinical setting	F – Teach in a community setting	G – Assess trainees	H – Evaluate course and undertake research in education	I – Assess & mentor other educators
	(1) Identify the targeted educational needs of the learner(s)	(1) Identify and direct learners to multimedia learning resources (texts, audiovisual resources, etc.) to support training and education	(1) Assist learners to reflect on their own experiences, strengths and areas for improvement through questioning, feedback and self-assessment	(1) Select and utilise teaching methodologies tailored to audience size and setting (problem-based learning, didactics, etc.)	(1) Teach physical exam and history-taking skills in both the educational and clinical environment	(1) Teach skills related to community-oriented primary care, including community engaged participatory assessment, planning and evaluation	(1) Choose appropriate assessment instrument for the setting and information/skills being evaluated	(1) Identify and employ a range of tools for evaluating courses	(1) Identify and employ a range of tools for evaluating educators
	(2) Define the learning outcomes expected from the proposed educational programme	(2) Design instructional resources, including handouts, handbooks, protocols and study guides	(2) Assist learners in developing their own individualised learning plans	(2) Identify and employ audiovisual aids effectively to ensure audience engagement	(2) Teach skills needed to find and apply evidence from the literature to clinical practice	(2) Challenge learners to consider how a community’s history (including exposure to colonialism or racism) may impact its health in the present	(2) Assess performance in a variety of settings (knowledge-based examinations, skills labs and performance in the clinical environment)	(2) Identify and employ a range of tools for evaluating resources materials	(2) Provide feedback to other educators on their individual and course evaluations
	(3) Design teaching strategies and learning experiences to match the outcomes	(3) Make appropriate use of clinical simulators for education	(3) Direct learners to locate relevant information in the learning environment, including clinical protocols and systems resources	(3) Organise and run video and teleconference e-learning sessions	(3) Teach clinical reasoning and decision-making skills in both the educational and clinical setting		(3) Produce and interpret learner profiles	(3) Integrate results of evaluations into quality improvement plans for educational programmes	(3) Mentor and coach other educators
	(4) Implement planned teaching		(4) Develop an environment where learners feel comfortable proactively giving feedback to faculty and colleagues		(4) Teach approaches to partnering with patients and colleagues in the clinical and practice environment, including demonstrating attitudes of compassion and respect and patient-centred care		(4) Set appropriate standards for learners at different levels of training and education	(4) Engage in research in medical education using sound educational research techniques	
			(5) Counsel, motivate and mentor learners in their growth as a learner and professional		(5) Teach strategies for practice management		(5) Assess applicants for admission to the educational programme		
					(6) Foster the learner’s sense of ownership for facilitating patient care				

**TABLE 4:** A model for family medicine faculty development: How the family medicine educator approaches their teaching.

Level	How the family medicine educator approaches their teaching *‘Doing the thing right’*				

Level 1	Knowledge of systems & principles			Interpersonal skills & professional approach	Analytical & research skills
Level 2	A – Understand the principles of education	B – Understand the principles of generalism	C – Understand the larger socio-political, legal, cultural and environmental context for learning, clinical medicine and community health	D – Demonstrate appropriate attitudes, ethical understanding and legal awareness	E – Cultivate knowledge of evidence base for education
	(1) Identify theories of learning, including principles of problem-based, cooperative and opportunistic learning	(1) Describe the unique role of generalists in the health system and education	(1) Identify teaching regulations that apply to the setting in which the educator works with learners	(1) Model intellectual humility and curiosity through openness to new ideas and critique	(1) Utilise the educational evidence base as the basis for teaching and learning strategies adopted
	(2) Identify and describes methods to support learners with different preferred learning styles	(2) Describe the value of a generalist to the patient, community and broader health system	(2) Describe grievance and disciplinary procedures in the teaching environment	(2) Face challenges and set-backs with humour and adaptability	(2) Develop familiarity with literature sources on medical education
	(3) Identify approaches to navigating small-group dynamics and principles of cooperative learning		(3) Demonstrate leadership within the cultural milieu	(3) Model enthusiasm for education and for the subject(s) being taught and appreciation for one’s role as an educator	
	(4) Describe principles of instructional design and curriculum planning		(4) Identify specific health challenges and social strengths in the community of practice	(4) Model a curiosity and interest in learning new educational technologies	
	(5) Identify principles and strategies for cultivating and assessing outcomes-based education		(5) Identify the policy and legal framework in which the health system operates	(5) Display respect for and show empathy and interest in learners.	
	(6) Describe the value of interdisciplinary education and describe approaches to fostering interprofessional learning		(6) Describe the One Health Framework	(6) Act on concerns and feedback from learners with confidentiality and impartiality	
	(7) Identify principles of assessment and feedback		(7) Prioritise health equity (ex. engage health systems and educational institutions to challenge inequitable policies and procedures)	(7) Consider and work to mitigate the risk of discrimination and bias in approach to learners, patients and colleagues	
	(8) Understand theories of change			(8) Model collaboration with practitioners of other clinical disciplines and professions	
	(9) Describe principles of evidence-based medicine			(9) Demonstrate respect for institutional goals	

**TABLE 5:** A model for family medicine faculty development: The family medicine educator as professional role model.

Level	The family medicine educator as a professional role model ‘The right person doing the thing’		

Level 1	Personal development	Modelling the role of a family doctor & educator	
Level 2	A – Engage in personal development with regard to teaching	B – Model the role of an educator within the teaching environment and health system	C – Model the role of a family physician through ongoing clinical activities & community engagement
	(1) Reflect upon and be aware of own strengths as a teacher	(1) Demonstrate competency with teaching responsibilities	(1) Model holistic thinking and the application of a biopsychosocial approach to clinical medicine and community health
	(2) Accept and respond to evaluation comments, constructive criticism, etc., from others	(2) Maintain an acceptable balance between service commitments, research and teaching, prioritising workload appropriately	(2) Model a commitment to generalism in clinical practice; role model excellent patient interaction and clinical exam, history-taking and management skills for patients across the lifespan; demonstrate comfort with clinical uncertainty
	(3) Seek and receive feedback from learners	(3) Describe the important role of the educator as researcher	(3) Model the ability to adapt to the specific resources and challenges of the local practice environment
	(4) Keep abreast of new teaching and learning techniques	(4) Model the role of a physician educator as a manager of teaching, including quality control	(4) Engage in ongoing reflection into personal strengths and weaknesses in clinical and community medicine
		(5) Model the role of a doctor as a teacher and learner within a multiprofessional team	(5) Demonstrate a commitment to life-long learning and improvements in clinical knowledge and community-engagement skills
		(6) Advocate for greater equity in policies and practice within educational and clinical settings	

**FIGURE 2:**
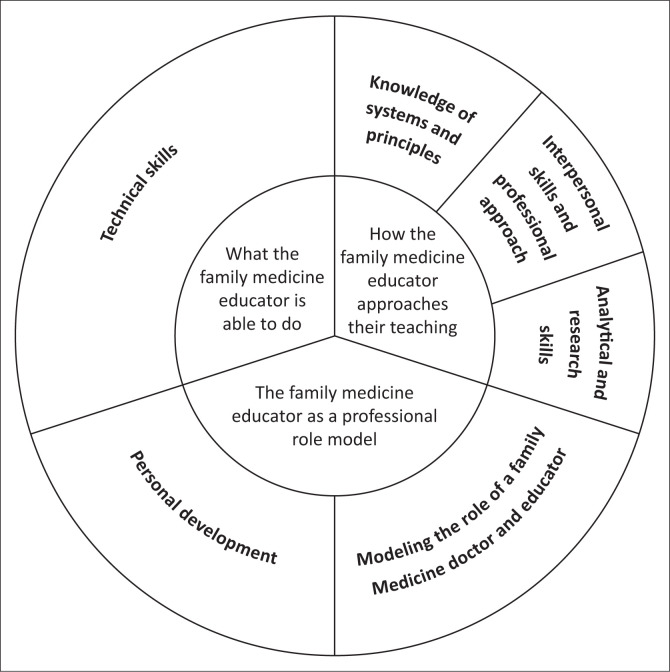
The key elements of family medicine educator faculty development.

The final model conceptualises the key elements of family medicine faculty development in three separate domains, ensuring that: (1) a family medicine educator is able to *do* the tasks required of an educator, (2) their approach is informed by knowledge of systems and principles of practice, incorporates professional skills and professionalism and reflects analytical skills and the ability to research effectively and (3) they effectively serve as a professional role model through their own roles as a clinician and educator and self-directed personal development (Figure 2).

## Discussion

Family medicine is a unique medical speciality for many reasons. Family medicine physicians serve as front-line primary care providers, capable of providing holistic, integrated, patient-centred, continuous care across the lifespan. In many regions of the world, family medicine physicians provide that care across a variety of settings, from outpatient clinics to emergency rooms, inpatient hospital rooms, intensive care units and obstetrical and surgical wards.

Beyond these clinical settings, family medicine physicians often play a public health role, working to address distal socioeconomic, cultural, environmental and political determinants of health in their communities. They do this through partnership with community leaders and public health teams. Because of the wide range of roles that family physicians play and the diverse settings in which they practise, a framework for faculty development for family medicine educators must incorporate unique skills that extend beyond those of clinical educators in other specialties. This is particularly true with emerging threats from climate change, such as disease outbreaks, that call for a versatile and adaptable workforce.

When asked what was missing from existing models for clinical educator faculty development, respondents highlighted the unique philosophical approach of family medicine. As a medical speciality grounded in the spirit of generalism, family medicine educators are uniquely positioned to provide clinical education informed by the principles of community and public health, contextualised in an understanding of health across the lifespan and whole person care.^25^

The family medicine faculty development experts whose expertise is reflected in this Delphi study represent this range of experience. Their alignment on the final elements of this framework suggests that the model is adaptable enough to be applied in different academic and community settings where family medicine education occurs. The framework is large and intentionally excludes prioritisation of elements, recognising that the prioritisation of different skills, knowledge, approaches and professional characteristics expected of the family medicine educator may differ depending on the setting in which they practise. In the United States and Canada, family medicine faculty development frameworks have been developed based on national priorities and needs. This new, globally developed framework offers a scaffold for family medicine academic units in a broad range of settings, facilitating prioritisation based on local/regional needs. We anticipate this will be particularly helpful as a starting point in places where no such framework exists.

This model outlines the philosophical tenets, technical components, and interpersonal competencies essential to family medicine educators, achieved through a gradual progression, from first focusing on technical skill acquisition to higher-level development of the educator as a role model. However, these may occur (and be facilitated) simultaneously in all three core areas.

Family medicine academic departments, community-based training programmes and individual practising family medicine preceptors and mentors in various settings across the globe may utilise this model to ensure quality and expertise. Educators may also utilise this framework as a tool for self-assessment to identify areas for needed professional development. Importantly, this framework is not intended to be a checklist of every aspect of knowledge, skills or expertise that every individual family medicine educator must have. The educators who train future family medicine physicians are as diverse as the speciality itself. A family medicine department or academic unit may utilise this framework to assess their entire team of educators and identify gaps in knowledge and/or expertise. Academic units and even family medicine professional associations may utilise this model to guide the creation of family medicine faculty development programming.

This study does have its limitations. The inclusion criteria limited participants to mid-to-late career family medicine faculty, excluding early career or recent family medicine graduates who may have been able to define their own needs better than existing ‘experts’. Additionally, there was some attrition of survey participants throughout the study, and it is possible that there was something unique about the knowledge and/or expertise of those participants. However, given the high response rate overall (ranging from 81% to 95.8%), we believe the impact of this is likely to be small.

## Conclusion

Family medicine is a speciality practised in diverse healthcare systems in a globally evolving healthcare landscape, characterised by variations and changes in practice and technology, patient expectations and epidemiological landscape. It is not well understood how these variations and changes may impact the optimal approach to train faculty who teach new family medicine practitioners. Considering this, a study of faculty development practices in family medicine around the world is not only timely, but also essential.

Frameworks provide a scaffold representing a common core of skills and values which may be contextualised for individual settings as needed. We believe this updated framework for family medicine faculty development promises to provide a valuable resource for educators, institutions and policymakers in the family medicine field, with the goal of enhancing the quality of care, driving innovation and meeting the healthcare needs of diverse populations worldwide.
